# The magnitude and correlates of common mental disorder among outpatient medical patients in Ethiopia: an institution based cross-sectional study

**DOI:** 10.1186/s13104-019-4394-x

**Published:** 2019-06-25

**Authors:** Mehbub Denur, Getachew Tesfaw, Zegeye Yohannis

**Affiliations:** 1 Worabe Comprehensive Specialized Hospital in SNNPR, Worabe, Ethiopia; 2 Department of Psychiatry, College of Medicine and Health Sciences, University of Gondar, Gondar, Ethiopia; 3 Research and Training Department, Amanuel Mental Specialized Hospital, Addis Ababa, Ethiopia

**Keywords:** Common mental disorders, Self-Reporting Questionnaire

## Abstract

**Objective:**

Common mental disorder has a high prevalence in the general population worldwide. One in four patients visiting any health services has at least one mental disorders and negatively impacts quality of life, physical wellbeing, poor level of functioning, and poor medication adherence. However, research into common mental illness and associated factors among people with outpatient medical patients in low and meddle-income countries is limited. Therefore, this study aimed to explore common mental disorder and associated factors among persons with outpatient medical illness in Ethiopia.

**Result:**

The prevalence of common mental disorder was found to be 39.2% with [95% CI 34.2%, 44.1%]. In the multivariate logistic regression, female sex [AOR: 2.03, 95% CI 1.28, 3.22], poor social support [AOR: 3.56 (95% CI 2.21, 5.73)], Diabetes mellitus [AOR: 5.25, 95% CI 2.35, 11.73], and substance use [AOR: 1.93, 95% CI 1.23, 3.04] were factors significantly associated with common mental disorder.

**Electronic supplementary material:**

The online version of this article (10.1186/s13104-019-4394-x) contains supplementary material, which is available to authorized users.

## Introduction

Common mental disorder (CMD) is a term used to describe a group of mental disorders that frequently include depression, anxiety, and somatoform disorders and it was highly prevalent in low and middle-income countries [1–3].

Over 450 million people were estimated to have a mental disorder and nearly one in four people meet criteria in their lives [1, 4]. The prevalence of CMD ranges from 14% nowadays to 15% by the year 2020 and the second leading cause of health disability in undeveloped countries [1, 5].

The disorders are more frequently observed in medical settings than in community settings and some studies showed that patients had seen at medical outpatient departments had up to 50% known to suffer from mental illnesses in addition to the physical disorders [6–8]. Depression and anxiety are quite common among patients at a primary care outpatient settings [9].

In Africa, CMDs are often misdiagnosed as physical illnesses because of many patients complained of somatic symptoms and mental illness had little attention in African countries [10]. In Ethiopia, the prevalence of CMD ranges from 23 to 58% in different medical settings and female sex, substance use, hypertension and diabetes mellitus, low income, and poor social support were factors associated with CMDs [11–15].

Risk factors for common mental disorder in the medical outpatient setting were gender, low income, substance use, chronic medical illness, and frequent doctor visits [1, 16]. Common mental disorders significantly impaired the patients quality of life, worsening their physical symptoms, and more likely to face ongoing stress [17]. Evidence on the prevalence of CMD and its intervention is the area of improvement among medical outpatient in developing countries [18]. However, research into CMDs and associated factors among people with outpatient medical patients in low and meddle-income countries are limited. Therefore, this study aimed to explore the magnitude of common mental disorder and determinates among people with medical patients in Ethiopia with a view of informing the development of interventions.

## Main text

### Methods

#### Study setting and design

An institution based cross-sectional study was conducted from May to June 2018 at WCSH Ethiopia. It is located in Southern Ethiopia and 173 km away from Addis Ababa, the capital of Ethiopia. It was established in 2014. It has 200 beds with 31,000 people at medical outpatient yearly visit at the movement.

#### Source population

All adult patients attended in the medical outpatient department.

#### Study population

All adult patients attended at a medical outpatient department, were included in the sample.

#### Inclusion criteria

All patients attended medical outpatient with an age of 18 years and above.

#### Exclusion criteria

Patients critically ill and on follow-up associated with psychiatric disorders were excluded.

#### Sample size determination

The sample size was calculated by using the single population proportion formula considering the following assumptions:

$$n = \frac{{\left( {Z\frac{\alpha }{2}} \right)^{2} p\left( {1 - p} \right)}}{{d^{2} }}$$

where n is the minimum sample size required for the study; Z is the standard normal distribution (Z = 1.96) with a confidence interval of 95% and ⍺ = 0.05; p = 58.6% prevalence of CMD at the University of Gondar hospital [19]. d is the absolute precision or tolerable margin of error (d) = 5% = 0.05; ${\text{n }} = \frac{{\left[ {\left( {1.96} \right)^{2} *0.586*\left( {1 - 0.586} \right)} \right]}}{{\left( {0.05} \right)^{2} }} = 373$. Then adding 10% (373 × 0.1 = 37.3 **≈** 38) of a non- responds total sample size for this study was 373 + 38 = 411.

#### Sampling techniques

The systematic sampling technique was used to select the study participants randomly. At the movement, 2583 medical outpatient are visited on monthly.

The sampling interval was determined by dividing to total study population who had follow-up during the data collection period by the total sample size; then the starting point was randomly selected.

#### Data collection

Data were collected using a pre-tested interviewer-administered questionnaire which contained CMD as the dependent variable and several other explanatory variables that included socio-demographic characteristics, clinical factors, social support, and substance use.

#### Measurement

Social support was assessed by the Oslo 3-item social support scale which had a 3-item questionnaire commonly used to assess social support and used in several studies. The sum score scale ranges from 3 to 14, and had three broad categories: “Poor support” 3–8, “moderate support” 9–11, and “strong support” 12–14 respectively [20]. It has been used in Ethiopia [21, 22].

Common mental disorders were measured by using Self-Reporting Questionnaires (SRQ-20), which is developed by a World Health Organization to screen CMD in a primary health care setting in low-income countries [23].

However the high illiteracy rate in Ethiopia and other countries of the same status, it was used in an interviewer-administered format, which was validated and subsequently used in clinical and community setting in Ethiopia. The scales used with cutoff score for CMD ≥ 8 [23] with the specificity and sensitivity were 83% and 89.5% respectively.

#### Data analysis

Data were entered into Epi-info 7 software after checking completeness and transferred to SPSS version 21 for analysis. Binary and multivariable logistic regression analyses were done to see the association of each independent variable with the outcome variable. The strength of the association was evaluated by using the adjusted odds ratio with a 95% CI and a p-value of less than 0.05 was considered statistically significant.

### Results

#### Socio-economic and demographic characteristics

A total of 406 respondents were included in the study with a response rate of 98.78%. The mean age of the participants was 36 (± 13.41) years. Out of the participants, 184 (45.3%) were between the ages of 18 and 30 years, almost half (51%) were male, and more than two-thirds (61.8%) were married. One hundred twenty-seven (31.3%) had a primary level education, 353 (86.9%) employed, and 248 (61.1%) came from a rural residence. The median monthly incomes were 1300 birr and ranging from 250 to 10,470 Ethiopian birr (Table 1).

**Table 1:** Distribution of adult patients by socio-demographic factors attending medical OPD at Worabe Comprehensive Specialized Hospital, 2018 (n = 406)

Variables	Categories	Frequency	Percent
Age in years	18–30	184	45.3
	31–40	92	22.7
	41–50	60	15
	> 50	70	17
Residence	Urban	158	38.9
	Rural	248	61.1
Gender	Male	207	51
	Female	199	49
Religion	Muslim	338	83.3
	Orthodox	56	13.8
	Protestant	12	3
Ethnicity	Silte	328	80.8
	Gurage	38	9.4
	Amhara	22	5.4
	Others	18	4.4
Marital status	Married	251	61.8
	Single	119	29.3
	Divorced	10	2.5
	Widowed	26	6.4
Educational status	Unable to read and write	112	27.6
	Primary education	127	31.3
	Secondary education	51	12.6
	College diploma	73	18
	Degree and above	43	10.6
Occupation	Employed	353	86.9
	Unemployed	53	13.1
Monthly income (birr)	< 1539	252	62.1
	≥ 1539	154	37.9

Others: Hadiya, Halaba, Oromo

Monthly income based on World Bank poverty level 1.9 $ per day

#### Clinical, social, and substance use characteristics

**Fig. 1:**
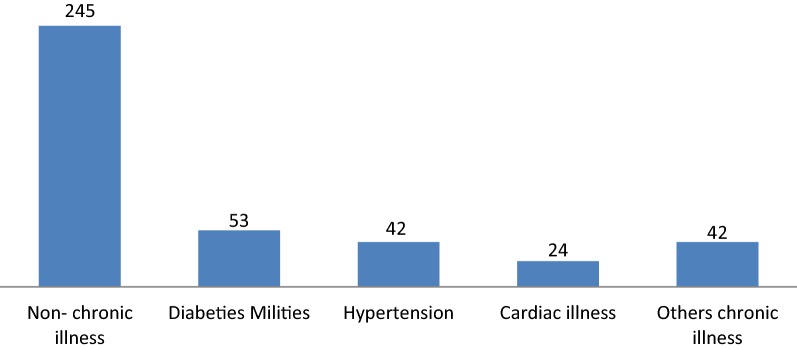
A distribution of current medical illness among participants attending WCSH medical outpatient department in 2018 (n = 406). Other chronic illness: chronic asthma, chronic kidney problem, both HTN and DM

Of the participants, 53 (13.1%) were on follow up with diabetes mellitus (Fig. 1), and two-thirds had ever visit the hospital (65.5%). Among participants, 28 (6.9%) had a family history of mental disorders and 17 (4.2%) had a personal history of mental disorder. At the movement, nearly half (47.3%) were taking substance. Regarding social factors, 150 (37%) and 48 (11.8%) of the participants had poor and moderate social support, respectively (Additional file 1).

#### Prevalence of CMD

This study showed that the prevalence of the common mental disorder among participants was 39.2%, with a 95% CI (34.2%, 44.1%).

#### Factors associated with CMD

To determine the correlations of independent variables with CMD, bivariate and multivariate binary logistic regression analyses were carried out. In bivariate analyses, factors including female sex, poor social support, diabetes mellitus, and current substance use were significantly associated with CMD at a p-value less than 0.05. These factors were entered into multivariate logistic regression model to control confounding effects.

By using multivariate logistic regression: female sex (AOR = 2.03, 95% CI 1.28, 3.22) was two times more likely to develop common mental disorders when compared to their counterparts. Poor social support was about 3.56 times more risky to CMD (AOR = 3.56, 95% CI 2.21, 5.57) when compared with good social support. Similarly, diabetes mellitus had five times more likely to have a CMD when compared to individuals had no chronic physical illness (AOR = 5.25, 95% CI (2.35, 11.73).

Current substance use (AOR = 1.93, 95% CI (1.23, 3.04) were associated with CMD, those who had a current use of substance were two times more likely to develop a CMD compared to individuals had no a current use of substances (Table 2).

**Table 2:** Bivariate and a multivariate logistic analysis result of the study subjects among adult patients attending medical OPD at Worabe Comprehensive Specialized Hospital, SNNR, Ethiopia 2018, (n = 406)

Variables	Categories	CMD		Crude OR (95% CI)	Adjusted OR (95% CI)
		Yes	No		
Gender	Male	69	138	1	1
	Female	90	109	1.65 (1.11, 2.47)	2.03 (1.28, 3.22)*
Age	18–30	62	122	0.57 (0.33, 0.99)	1.23 (0.58, 2.62)
	31–40	38	54	0.79 (0.42, 1.48)	1.25 (0.57, 2.72)
	41–50	26	34	0.86 (0.43, 1.72)	1.31 (0.59, 2.92)
	> 50	33	37	1	1
Duration of illness (months)	≤ 12	126	213	1	1
	>12	33	34	1.64 (0.97, 2.78)	0.97 (0.48, 1.96)
A family history of psychiatric illness	Yes	15	13	1.87 (0.87, 4.10)	2.03 (0.86, 4.81)
	No	144	234		1
Chronic illness	Diabetes mellitus	36	17	4.80 (2.53, 9.08)	5.25 (2.35, 11.73)*
	Hypertension	21	21	2.28 (1.17, 4.40)	1.78 (0.75, 4.22)
	Cardiac illness	10	14	1.62 (0.69, 3.81)	1.96 (0.70, 5.51)
	Others	17	25	1.54 (0.79, 3.02)	1.36 (0.56, 3.30)
	No-chronic illness	75	170	1	1
Social support	Poor	86	64	3.65 (2.34, 5.70)	3.56 (2.21, 5.57)*
	Moderate	17	31	1.49 (0.77, 1.90)	1.79 (0.86, 3.70)
	Strong	56	152	1	1
Current substance use	Yes	91	101	1.93 (1.29, 2.90)	1.93 (1.23, 3.04)*
	No	68	146	1	1

Others: chronic asthma, kidney problem, both HTN, and DM

p-value statically significant; p-value * < 0.05; p-value Hosmer and Lemeshow = 0.67

## Discussion

In the current study, the prevalence of CMD and its possible association with various factors was assessed. The results showed that a remarkable proportion of people with the outpatient medical department had CMD. The prevalence of CMD among patients with the outpatient medical department was found to be 39.2%.

Regarding prevalence, our result is in line with those of other studies carried out in Ethiopia, Kenya, Uganda, Qatar, and India reported the magnitude of common mental disorder to be 34.6%, 42%, 42%, 36.6%, and 38.6%, respectively [3, 5, 24–26].

On the other hand, our finding is higher than those of the studies done in two areas of Ethiopia among malaria and glaucoma patients in Jimma and Addis Ababa, and Tanzania, in the prevalence was estimated at 24.5%, 23.2%, and 24%, respectively [27–29]. The variations may be due to distinctions in sample sizes, measurement tools, population difference, and cutoff point variations to assess CMD. Only 300 participants were used to assess by using the same tool but the difference is the cutoff point used ≥ 11 in Jimma Ethiopia among malarias patients, but in Addis Ababa, Ethiopia among glaucoma patients by using the same instrument but the cutoff point greater than or equal to eleven might be their difference [27, 28]. In Tanzania, only 178 participants were assessed by using Clinical Interview Schedule-Revised (CIS-R) with cutoff points greater than 12 [29].

However, the present study finding was lower than those of other studies conducted in South Africa, in which the prevalence of the common mental disorder among chronic medical patients was 49.7% by using the Kessler-10 scale [30]. In Kuwait, only 100 participants were included their study among medical outpatients by assessing International Disease Classification-10 (ICD-10), in which the magnitude of CMD was 50% [11], and in Canada, the prevalence of CMD among 75 medical patients were 57.3% by assessing two tools Patient Health Questionnaire-9 (PHQ-9) and General Anxiety Disorder-7 (GAD-7) [31].

Female sex had two times more likely to develop a CMD compared to men. This increased prevalence of CMD might be due to hormonal and physiological mechanisms, increased responsibilities such as child rearing, care for other family members, a pressure of their roles, and responsibility in their community, gender discrimination, and related violence contribute to their poor mental health [32]. This was consistent with those other the studies in two areas of Ethiopia [19, 27].

Poor social support was about 3.56 times more risky for CMD than good support. This might be due to feeling isolation or decreased self-esteem, lack of social support, and somatic illness may lead to increased CMD [33]. This was supported by those of other studies were done among TB and hypertensive patients in Ethiopia [34, 35].

Patients with diabetes mellitus were associated with a CMD, which was five times more likely to develop CMD compared to those who did not chronic medical illness. A common mental disorder increased the risk of developing non-communicable disease especially diabetes mellitus is highly co-morbid with CMD [30]. Metabolic components like fasting blood glucose were independently associated with anxiety [36]. It was supported by two other studies conducted among patients with glaucoma and hypertension in Addis Ababa, Ethiopia [27, 35].

Current use of a substance was significantly associated with a CMD, which was two times more likely to develop a common mental disorder as compared those who had no a current use of a substance, this was supported by the study among patients with TB in Ethiopia [34]. This might be due to the patients with a common mental disorders were more prone to use substance to relieve stress or anxiety symptoms associated with medical problems [37]. Substance use was strongly linked with the CMD [10, 16].

### Conclusions

The prevalence of CMD was ground to be high. This study confirmed that patients attending a medical outpatient department had a negative impact on the mental health of affected individuals. Female sex, poor social support, diabetes mellitus, and current substance use were factors significantly associated with a CMD. The Ministry of health should develop guidelines to screen and treat a common mental disorder among patients in adult medical outpatient departments. Further research on risk factors of CMD in medical outpatient departments should be conducted to strengthen and broaden these findings.

## Limitation

A cross-sectional design cannot permit conclusions for some variables, for example, to decide whether CMD symptoms are risk for or a consequence.

This finding is likely only to hint at the complex interactions between common mental disorder and explanatory variables (risk factors).

## Additional file

**Additional file 1.** Distribution of patients by clinical, social, and substance factors attending medical OPD at Worabe Comprehensive Specialized Hospital, 2018 (n = 406). Of the participants, 53 (13.1%) were to follow up with diabetes mellitus (Fig. 1), and two-thirds had ever visited the hospital (65.5%). Among the participants, 28 (6.9%) had a family history of mental disorders and 17 (4.2%) had a personal history of mental disorder. At the movement, nearly half (47.3%) was taking the substance. Regarding social factors, 150 (37%) and 48 (11.8%) of the participants had poor and moderate social support, respectively.

## Data Availability

The dataset during and/or analyzed during the current study available from the corresponding author on reasonable request.

## Abbreviations

AOR: adjusted odds ratio
CMD: common mental disorder
COR: crude odds ratio
GHQ: General Health Questionnaire
OR: odds ratio
OPD: outpatient department
SNNPR: Southern Nation Nationalities Peoples Region
SRQ: Self-Reporting Questionnaire
SPSS: Statistical Package for Social Sciences
TB: tuberculosis
UoG: University of Gondar
WCSH: Worabe Comprehensive Specialized Hospital
WHO: World Health Organization
