# Supplementary material for: The magnitude and correlates of common mental disorder among outpatient medical patients in Ethiopia: an institution based cross-sectional study
Source: BMC Res Notes. 2019 Jun 25;12:360. doi: 10.1186/s13104-019-4394-x (PMC6593598; doi:10.1186/s13104-019-4394-x)
Supplement: Supplementary file 1 — Additional file 1. Distribution of patients by clinical, social, and substance factors attending medical OPD at Worabe Comprehensive Specialized Hospital, 2018 (n = 406). Of the participants, 53 (13.1%) were to follow up with diabetes mellitus (Fig. 1), and two-thirds had ever visited the hospital (65.5%). Among the participants, 28 (6.9%) had a family history of mental disorders and 17 (4.2%) had a personal history of mental disorder. At the movement, nearly half (47.3%) was taking the substance. Regarding social factors, 150 (37%) and 48 (11.8%) of the participants had poor and moderate social support, respectively. [file 13104_2019_4394_MOESM1_ESM.docx]

Additional file 1: Distribution of patients by clinical, social, and substance factors attending medical OPD at Worabe Comprehensive Specialized Hospital, 2018 (n=406)

| Variables | Categories | Frequency | Percent |
| --- | --- | --- | --- |
| Duration of medical illness | ≤ 12 months | 339 | 83.5 |
|  | > 12 months | 67 | 16.5 |
| Ever visit of hospital | No | 140 | 34.5 |
|  | Yes | 266 | 65.5 |
| A family history of mental illness | No | 378 | 93.1 |
|  | Yes | 28 | 6.9 |
| Personal history of mental illness | No | 389 | 95.8 |
|  | Yes | 17 | 4.2 |
| Current substance use | Yes | 192 | 47.3 |
|  | No | 214 | 52.7 |
| Social support | Poor | 150 | 37 |
|  | Moderate | 48 | 11.8 |
|  | Good | 208 | 51.2 |
